# Talin Contains A C-Terminal Calpain2 Cleavage Site Important In Focal Adhesion Dynamics

**DOI:** 10.1371/journal.pone.0034461

**Published:** 2012-04-04

**Authors:** Neil Bate, Alexandre R. Gingras, Alexia Bachir, Rick Horwitz, Feng Ye, Bipin Patel, Benjamin T. Goult, David R. Critchley

**Affiliations:** 1 Department of Biochemistry, University of Leicester, Leicester, United Kingdom; 2 Department of Cell Biology, Univeristy of Virginia School of Medicine, Charlottesville, Virginia, United States of America; 3 Department of Medicine, University of California San Diego, La Jolla, California, United States of America; Kings College London, United Kingdom

## Abstract

Talin is a large (∼2540 residues) dimeric adaptor protein that associates with the integrin family of cell adhesion molecules in cell-extracellular matrix junctions (focal adhesions; FAs), where it both activates integrins and couples them to the actin cytoskeleton. Calpain2-mediated cleavage of talin between the head and rod domains has previously been shown to be important in FA turnover. Here we identify an additional calpain2-cleavage site that removes the dimerisation domain from the C-terminus of the talin rod, and show that an E2492G mutation inhibits calpain cleavage at this site in vitro, and increases the steady state levels of talin1 in vivo. Expression of a GFP-tagged talin1 E2492G mutant in CHO.K1 cells inhibited FA turnover and the persistence of cell protrusion just as effectively as a L432G mutation that inhibits calpain cleavage between the talin head and rod domains. Moreover, incorporation of both mutations into a single talin molecule had an additive effect clearly demonstrating that calpain cleavage at both the N- and C-terminal regions of talin contribute to the regulation of FA dynamics. However, the N-terminal site was more sensitive to calpain cleavage suggesting that lower levels of calpain are required to liberate the talin head and rod fragments than are needed to clip off the C-terminal dimerisation domain. The talin head and rod liberated by calpain2 cleavage have recently been shown to play roles in an integrin activation cycle important in FA turnover and in FAK-dependent cell cycle progression respectively. The half-life of the talin head is tightly regulated by ubiquitination and we suggest that removal of the C-terminal dimerisation domain from the talin rod may provide a mechanism both for terminating the signalling function of the talin rod and indeed for inactivating full-length talin thereby promoting FA turnover at the rear of the cell.

## Introduction

Cell migration involves a complex cycle of inter-related events initiated by extracellular cues that establish cell polarity and membrane protrusion at the leading edge driven by actin polymerisation [1]. This is followed by the assembly of small highly dynamic nascent adhesions, a fraction of which mature into larger more stable structures, the subsequent translocation of the cell body, and the detachment of the trailing edge [2]. The migratory cycle is orchestrated from within the cell by the Rho-family of GTPases, which regulate both actin polymerisation and the architecture and dynamic properties of the newly assembled actin filaments, as well as myosin II-dependent contractility [3], [4], [5].

Cell-extracellular matrix interactions are typically mediated by members of the integrin family of transmembrane αβ-heterodimers, and both “inside-out" and “outside-in" signalling [6] can trigger the formation of multi-protein complexes on the cytoplasmic face of integrins that are important in cell adhesion and migration [7], [8], [9]. One of the key proteins required for the assembly of cell-matrix adhesions is the adaptor protein talin [10], [11], which can bind both integrins and F-actin, and can also switch integrins from a low to high affinity state [12], [13]. Most cells express two closely related talin isoforms [14], and cells depleted of talin1 assemble far fewer FA and show reduced cell spreading and migration [15], although this phenotype can be rescued by talin2 [16], [17]. Moreover, talin1 is required to form the slip bond between fibronectin/integrin complexes and the actomyosin contractile apparatus within the cell [18].

**Figure 1:**
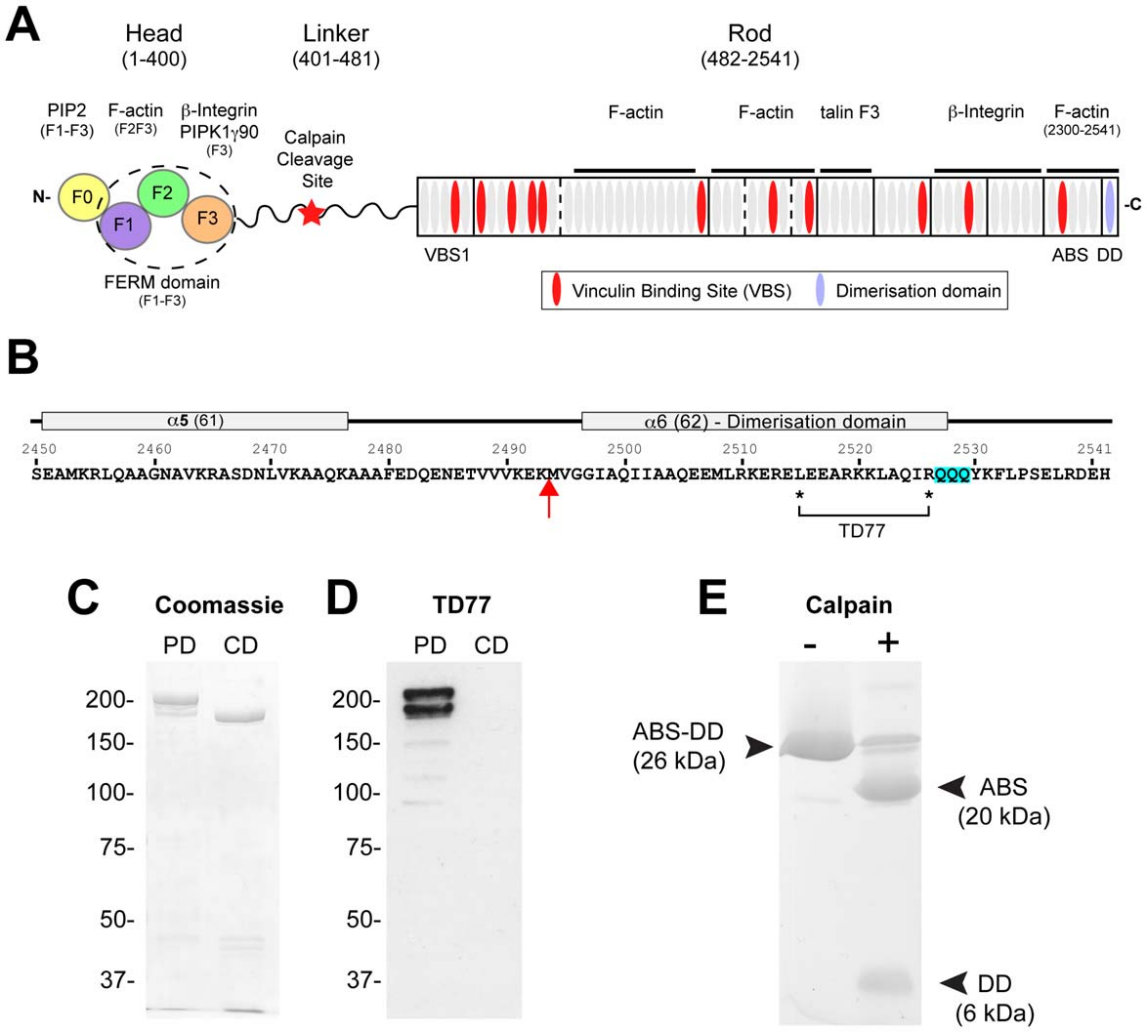
The talin1 C-terminal dimerisation domain is clipped off by calpain2. (A) Talin consists of an N-terminal head (residues 1–400) containing an atypical extended FERM domain (made up of F0–F3 domains) and a flexible rod containing 61 α-helices (grey elipses) organised into 13 helical bundles and terminating in a single helix responsible for dimerisation (DD). The position of various ligand-binding sites are shown including the C-terminal actin-binding site (ABS). The head and rod are joined by a linker region that is cleaved by calpain2 between Q433 and Q434. (B) Sequence and secondary structure of the last two helices in the talin rod. The two asterisks indicate residues L2515 and R2526, key determinants of the epitope recognised by the TD77 monoclonal antibody. The red arrow indicates the calpain2 cleavage site. (C,D) Purified turkey gizzard talin was incubated with calpain2 to generate a partial digest (PD) and a complete digest (CD). Cleavage products were resolved by SDS-PAGE and stained either with (C) Coomassie blue or (D) the monoclonal antibody TD77 that recognises the DD. (E) A talin polypeptide (residues 2300–2541) containing the C-terminal ABS and DD domain was incubated with calpain2 in the presence (+) or absence (−) of calcium. N-terminal sequencing and mass spectroscopy of the products show that the largest band corresponds to the ABS (2300–2493) and the smallest fragment (M2494) corresponds to the dimerisation domain (2494–2541).

Talins (∼270 kDa; ∼2540 amino acids) are comprised of an N-terminal head (1–400) containing an atypical FERM domain [19] with binding sites for β-integrin tails [20], [21], F-actin [22], the type 1 isoform of PIPKγ [23], [24] and acidic phospholipids such as PIP2 [25], [26] (Fig. 1A). The talin head is linked via residues 401–481 to a long flexible rod (482-C-terminus) consisting of 61 α-helices organised into a series of amphipathic 4- or 5-helix bundles [27], [28], [29]. The talin rod contains an integrin binding site [30], [31] and several actin-binding sites (ABS) [32], the best characterised of which is associated with the most C-terminal helical bundle [27]. This is followed by a single helix (helix 62) that forms an anti-parallel dimer, and appears to be largely responsible for formation of talin homodimers [27]. Interestingly, talin dimerisation is essential to the activity of the C-terminal ABS, which binds along the surface of a single actin filament. The other notable feature of the talin rod is that it contains several binding sites for vinculin [33], which itself has numerous binding partners [34], and is thought to stabilise FA [35], [36], [37].

While talin plays a prominent role in FA assembly, calpain2-mediated cleavage of talin [38], [39] and a number of other FA proteins [40], [41], [42] including β3-integrin tails [43] is thought to be important in the disassembly of FA and LFA-1 adhesions in T-cells [44], and for the mesenchymal though not the amoeboid form of cell migration [45]. Calpain2 was originally shown to cleave talin between residues Q433 and Q434 in the region between the head and rod domains [46], and mutation of L432G rendered talin partially resistant to cleavage and suppressed the turnover of talin as well as the FA proteins paxillin and zyxin [38]. This suggests that calpain2 cleavage of talin is an important step in FA turnover. More recent data shows that the talin head liberated by calpain2 cleavage is itself crucial to FA turnover and cell spreading, and its half-life is tightly regulated by a balance between Smurf1-mediated ubiquitination and proteasomal destruction versus cdk5-mediated S425 phosphorylation which inhibits Smurf1 binding [47]. However, the fate of the talin rod has not been extensively studied, although in platelets, it is recruited with αIIbβ3-integrin into the TritonX100 resistant cytoskeletal fraction while the talin head was Triton soluble [48].

Here we report the identification and characterisation of a second calpain2 cleavage site in talin that removes the dimerisation helix at the C-terminus of the talin rod. Expression of GFP-talin carrying mutations in this site singly or in combination with the L432G mutation markedly suppressed protrusion persistence and FA turnover suggesting that both sites are important in regulating the dynamic properties of FA.

## Results

### Characterisation of a novel calpain2-cleavage site in the talin rod

It is well documented that calpain2 cleaves talin1 in the linker region between the head and rod domain (Fig. 1A), an event that is important in FA turnover and cell migration [38]. The cleavage site has been mapped to between Q433 and Q434 (STVLQ-QQYNR) [46]. Interestingly, there is a second QQQ sequence in talin1 close to the C-terminus of the talin rod (residues 2527–2529) just downstream of the dimerisation helix (Fig. 1B). We therefore considered the possibility that calpain2 might cleave talin1 at this C-terminal site. To address this, we used a monoclonal antibody TD77, that we have previously shown recognises an epitope towards the C-terminal region of talin [49]. Western blotting showed that TD77 binds to a purified recombinant mouse talin1 polypeptide spanning residues 2300–2541 that contains both the C-terminal actin-binding site and the dimerisation domain, but not to a polypeptide (2300–2482) lacking the dimerisation domain (Fig. S1). The epitope for TD77 was further characterised by testing a series of point mutations in the C-terminal helix [27]. The data show that residues L2515 and R2526 are key determinants of TD77 binding (Fig. S1).

We then used TD77 to monitor calpain2-cleavage of purified turkey gizzard talin. Coomassie stained gels of partial talin digests showed two bands (Fig. 1C), both of which were recognised by TD77 (Fig. 1D) and therefore contain the dimerisation domain. We conclude that the upper band is intact talin and the lower band, the talin rod liberated by calpain2 cleavage in the linker between the talin head and rod. As expected, intact talin completely disappeared following a more prolonged incubation with calpain2, but interestingly, it was replaced by a Coomassie-stained polypeptide that was slightly smaller than the TD77-positive rod fragment liberated by partial cleavage (Fig. 1C). Significantly, this fragment was not recognised by TD77 (Fig. 1D) suggesting that calpain2 can indeed cleave the last helix from the talin rod domain.

To map the C-terminal calpain2 cleavage site more precisely, we used the purified recombinant talin1 2300–2541 polypeptide which has an apparent molecular weight of ∼25 kDa by SDS-PAGE. Incubation of this polypeptide with calpain2 in the presence, but not the absence of calcium, generated a proteolytic fragment of ∼20 kDa (Fig. 1E). If cleavage had occurred as predicted, between residues 2527-QQQ-2529, only 13 residues would have been removed (Fig. 1B), and the molecular weight of the proteolytic fragment would have been much larger. N-terminal sequencing of the ∼20 kDa fragment showed that it had the same sequence as the uncleaved polypeptide, indicating that cleavage must take place towards the C-terminus. Careful inspection of the cleavage products resolved by SDS-PAGE revealed a small proteolytic fragment running close to the bottom of the gel (Fig. 1E) the N-terminus of which was M2494 (Fig. 1B). Cleavage at this site would release a 5.7 kDa C-terminal fragment, consistent with the shift observed in the molecular weight of the 2300–2541 talin1 polypeptide following calpain2 cleavage. In summary, we have identified a novel calpain2 site between residues K2493-M2494 in the talin rod that clips off the dimerisation domain.

### Both the N- and C-terminal calpain2 cleavage sites in talin1 are in unstructured regions

**Figure 2:**
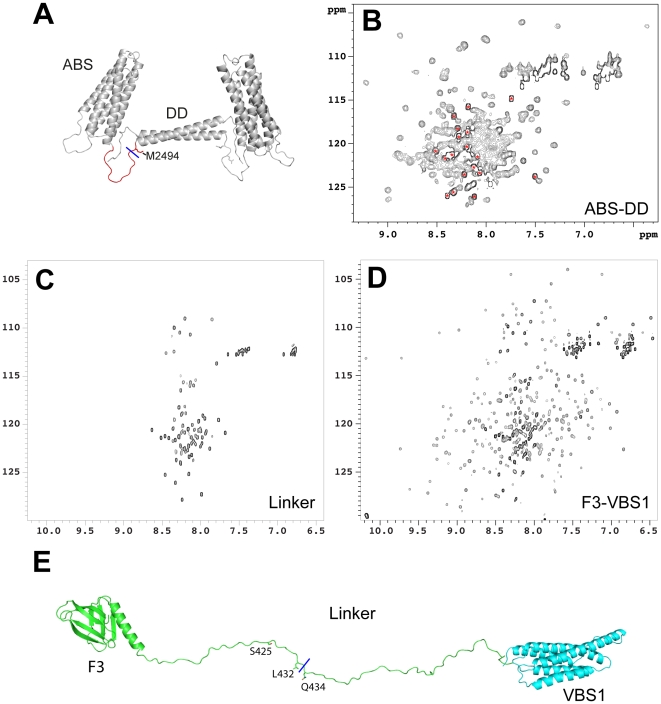
Both calpain cleavage sites are located in predominantly unstructured regions. (A) The structures of the C-terminal ABS and the dimerisation domain of talin1 are incorporated into a model of the C-terminal region of the protein. The calpain2 cleavage site is indicated (blue line). (B–D) ^1^H,^15^N HSQC spectra of the following talin1 polypeptides (150 µM); (B) Residues 2294–2541 spanning the C-terminal ABS and dimerisation domain (DD). The intense poorly dispersed peaks (red asterisks) relate to the 18 amino acid linker between the ABS and the DD (as deduced by comparison of the spectra of residues 2294–2491 and 2300–2482), and show that the linker is predominantly disordered. (C) Residues 400–480 - the linker between the talin1 head and rod that contains the calpain-cleavage site. The intense poorly dispersed signals in the centre of the spectrum indicate that the linker is predominantly unstructured. (D) Residues 309–655 containing the F3 FERM domain, the linker and the VBS1 rod domain. (E) A model of the linker region generated using MODELLER [50], [51] showing that it can span ∼200 Å when fully extended. The calpain2 cleavage site (blue line) between Q433–Q434 is shown along with L432, which when mutated to a glycine, reduces calpain2 cleavage. The CDK5 phosphorylation site (S425) that regulates binding of Smurf1 [47] is also shown.

We have previously determined the structure of the C-terminal region of talin1 [27]. The NMR structure of residues 2300–2476 shows a 5-helix bundle (the THATCH core domain) while the crystal structure of residues 2497–2527 reveals a single helix that forms an anti-parallel dimer, and is responsible for talin1 dimerisation (Fig. 2A). However, we were unable to determine the structures of a segment of ∼20 residues between the 5-helix bundle and the dimerisation domain, and a stretch of 14 residues at the extreme C-terminus of talin1 (Fig. 1B). Analysis of the NMR spectra of various C-terminal talin polypeptides suggests that the 5-helix bundle and the dimerisation domain interact [27], but also that the majority of the residues between the two domains are largely unfolded (Fig. 2B). Thus, calpain2 cleaves in an unstructured region between the THATCH core domain and the dimerisation helix.

The calpain2 cleavage site in the linker region (401–481) between the talin F3 FERM domain (residues 309–400) and the 5-helix bundle, VBS1 (482–655) at the start of the talin rod (Fig. 1A) is also predicted to be largely unstructured, although this has not been investigated directly. We therefore used NMR to analyse a recombinant ^15^N-labelled 400–480 talin1 polypeptide. The ^1^H-^15^N HSQC spectrum was consistent with a peptide that is predominantly unstructured with low signal dispersion in the proton dimension (Fig. 2C). Comparison of the spectra of 400–482 with the larger construct 309–655 (which contains the two flanking domains F3 and VBS1) showed that many of the signals from the linker region are at identical positions in the two spectra (Fig. 2D), confirming that even in the context of larger talin polypeptides, this linker region remains unstructured. We modelled the linker region in the context of the talin1 309–655 polypeptide using the Modeller software [50], [51]. Strikingly, the linker region has the potential to extend ∼200 Å equivalent to approximately four talin rod helical bundle domains (Fig. 2E). In summary, it is apparent that calpain2 cleaves talin in two unstructured regions at the N-terminal and the C-terminal region of the protein.

### Relative sensitivities of the N- and C-terminal calpain2 cleavage sites in talin1 and talin2

**Figure 3:**
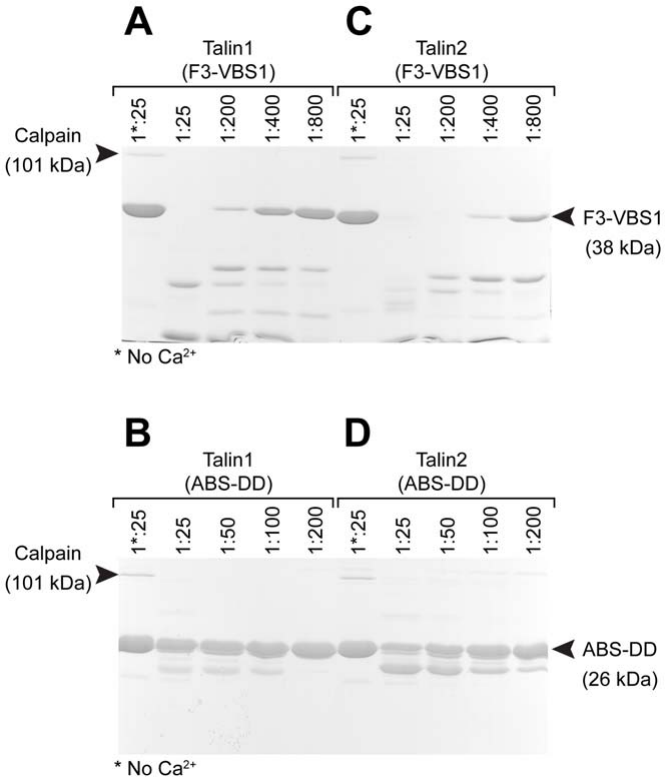
The C-terminal calpain2 site in talin1 is less sensitive to cleavage than that in the linker between the head and rod. (A) A talin1 F3-VBS1 polypeptide (residues 309–655) containing the linker between the head and rod and (B) a talin1 polypeptide containing the C-terminal ABS and DD (residues 2300–2541) were incubated with varying dilutions of calpain2. Cleavage products were analysed by SDS-PAGE and visualised with Coomassie blue. The N-terminal calpain2 cleavage site in talin1 is more sensitive than the C-terminal site. (C, D) Calpain2 cleavage of the equivalent talin2 polypeptides. Both talin2 polypeptides were somewhat more sensitive to calpain2 cleavage than the corresponding talin1 polypeptides.

We next compared the relative sensitivities of the N- and C-terminal regions of talin1 to calpain2 cleavage. Interestingly, the N-terminal F3-VBS1 (residues 309–655) talin1 polypeptide was significantly more sensitive to cleavage than the C-terminal polypeptide spanning the C-terminal ABS and DD (residues 2300–2541) (Fig. 3A,B). Even at a calpain: talin1 ratio of 1∶200, the N-terminal polypeptide was quantitatively converted to smaller fragments while only a small percentage of the C-terminal polypeptide was cleaved under these conditions. This suggests that the N-terminal calpain2 site might be more exposed than that at the C-terminus.

Although talin1 and 2 are highly conserved proteins (74% identity), the sequence in the vicinity of the N-terminal calpain2 cleavage site differs, and it has been suggested by Senetar et al. that this may confer different sensitivities to calpain2 cleavage [52]. Indeed, Western blots of muscle extracts showed two large talin1 immuno-reactive proteins thought to represent intact talin1 and the talin1 rod, while talin2 blots showed only a single band thought to be full-length protein. On this basis, the authors propose that talin2 may be more resistant to calpain2 cleavage than talin1. We therefore compared the relative sensitivity of N- and C-terminal talin1 and talin2 polypeptides to calpain2 cleavage. The results do not bear out the above suggestion, and both the N- and C-terminal talin2 polypeptides were somewhat more sensitive to calpain2 cleavage than their talin1 counterparts (Fig. 3C,D). Perhaps the lack of talin2 cleavage in cell extracts is more a reflection of lack of exposure of the calpain2 cleavage sites rather than its inherent susceptibility to calpain2 cleavage.

### Identification of mutations that block calpain2 cleavage in vitro and in vivo

**Figure 4:**
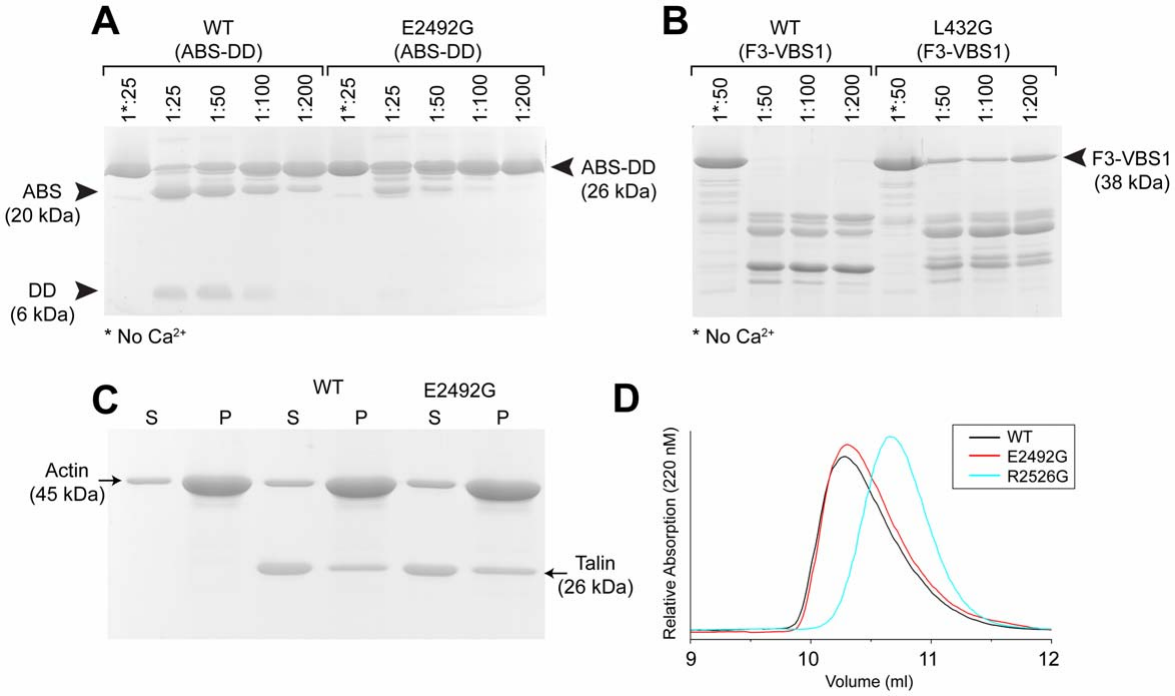
An E2492G mutation in talin1 2300–2541 reduces sensitivity to calpain2 cleavage but does not affect actin binding or dimerisation. (A) Wild-type talin 2300–2541 (ABS-DD) and an E2492G mutant were incubated with varying dilutions of calpain2, and their sensitivity to cleavage analysed by SDS-PAGE. (B) For comparison, the same experiment was conducted with wild-type talin 309–655 (F3-VBS1) and a L432G mutant in the linker between the head and rod which has previously been shown to partially inhibit calpain2 cleavage [38]. (C) Actin co-sedimentation assay using wild-type talin 2300–2541 and the E2492G mutant. Comparable amounts of each protein co-sedimented with F-actin (Pellet (P); Supernatant (S). Binding was quantified using imageJ analysis of the relevant bands; wild-type 100±2.3 versus mutant 105±4.3. (D) Gel filtration of wild-type talin1 2300–2541 and the E2492G mutant show that both form dimers. The monomeric R2526G mutant [27] is shown for comparison.

To evaluate the importance of the C-terminal calpain2 cleavage site in talin1 in a cellular context, we first sought to identify mutations that suppress cleavage. Previous studies have shown that substituting the residue at the P2 position upstream of the calpain cleavage site partially inhibits calpain cleavage [38], [53], and we therefore introduced an E2492G mutation into a talin 2300–2541 polypeptide. This mutation proved to significantly reduce the sensitivity of the C-terminal talin1 polypeptide to calpain2 cleavage (Fig. 4A). Indeed, it was substantially more effective in suppressing cleavage than the L432G mutation in the linker between the talin head and rod (Fig. 4B). Importantly, the E2494G mutation did not affect the ability of the C-terminal talin polypeptide to bind F-actin (Fig. 4C) or to dimerise (Fig. 4D), while a R2526G mutation in the dimerisation helix has been shown to inhibit both talin dimerisation (Fig. 4D) and actin binding [27].

**Figure 5:**
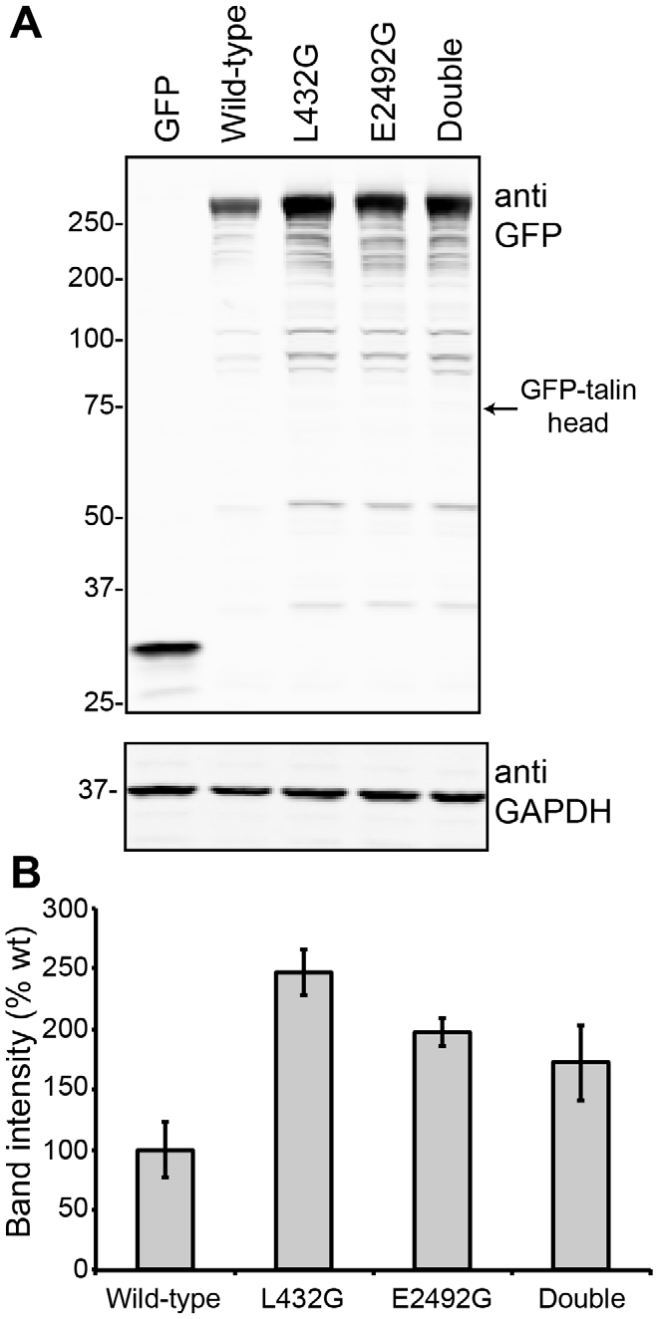
GFP-talin1 calpain2-resistant mutants accumulate in HEK293 cells. (A) Western blots (anti-GFP) of cells transfected with the following constructs: GFP alone; GFP-talin1; GFP-talin1(L432G); GFP-talin1 (E2492G); a GFP-talin1(L432G,E2492G) double mutant. Anti-GAPDH was used as a loading control. GFP-talin1 head (arrow) was not detected in these cells. (B) Band intensity was quantified using the Odyssey software (LI-COR). Bars indicate SEM of triplicate determinations. Essentially similar results were obtained in three separate experiments.

To establish whether mutations in the N- and C-terminal calpain cleavage sites affect the stability of talin1 *in vivo*, we transfected HEK293 cells with wild-type GFP-tagged talin1 or the L432G or E2492G calpain-resistant mutants as well as a L432G, E2492G double mutant. Western blotting for GFP showed that both the L432G and the E2492G calpain-resistant talin1 mutants accumulated to a significantly greater extent than wild-type GFP-talin1 (Fig. 5) indicating that both calpain sites are important in talin1 turnover, although the GFP-talin1 double mutant was no more stable than GFP-talin1 carrying the single mutations.

In an attempt to identify the C-terminal talin1 helix liberated by calpain2 cleavage, we expressed doubly tagged GFP-talin1-mCherry in HEK293 cells. However, we were unable to detect the C-terminal talin1 helix fused to mCherry by Western blotting (data not shown). Similarly, we were unable to detect GFP-talin1 head in cells transfected with GFP-talin1 (Fig. 5A). The talin head liberated by calpain2 cleavage is ubiquitinated and rapidly degraded by the proteasome [47], and similar mechanisms may lead to clearance of the C-terminal talin1 polypeptide.

### Effects of calpain2-resistant talin mutants on adhesion dynamics

**Figure 6:**
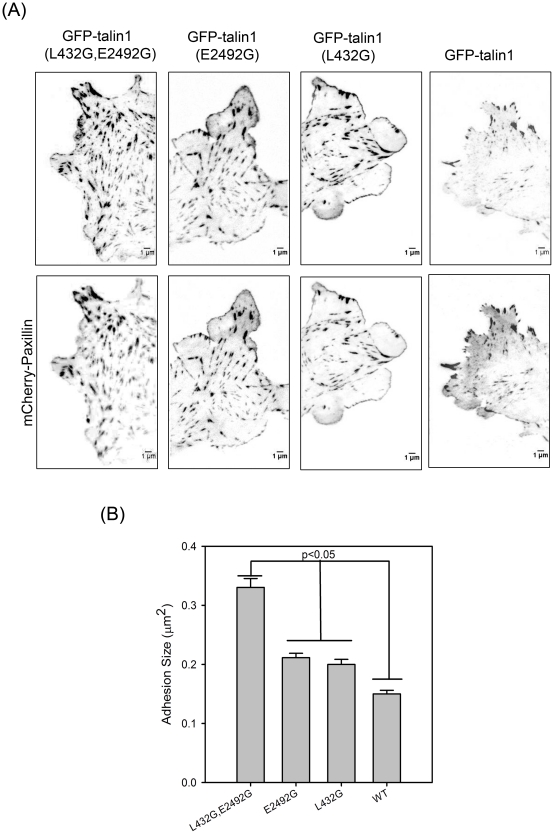
Size of adhesions in CHO.K1 cells expressing GFP-talin1 mutants. (A) TIRFM images of CHO.K1 cells co-expressing paxillin-mCherry and the GFP-talin1 mutants imaged 4 hr after plating on 2 µg/ml FN. An increase in adhesion size is observed in cells expressing the GFP-talin1 double mutant (L432G, E2492G) compared to cells expressing either the GFP-talin1 L432G or GFP-talin1 E2492G mutants as indicated in the box plot in (B). The Dunn's method was used to test the statistical significance of the pairwise differences in the distributions. The number of adhesions (Nadh) and cells (Ncells) included in the analysis was 182, 6 (L432G, E2492G); 192,5 (L432G); 195,6 (E2492G); 222,7 (WT) respectively. Error bars are SEM.

To investigate the possible significance of calpain2-mediated proteolysis of talin at both the N- and C-terminal sites, we co-transfected CHO.K1 cells with paxillin-mCherry, and one of three GFP-talin1 mutants i.e. (L432G), (E2492G), and (L432G, E2492G). Using TIRF microscopy on cells with similar, low, expression levels, we found that all of the various GFP-talin1 mutants localized to focal adhesions (FA) (Fig. 6A). However, the size of the adhesions varied among the different mutants, and there was an increase in the number of larger adhesions in cells expressing the GFP-talin1 double mutant when compared to the single point mutants or wild-type talin1 (Fig. 6B). Under these experimental conditions, the adhesions were relatively stable and disassembled on the time scale of tens of minutes. These observations agree with Franco et al. using the L432G talin1 mutant [38]

**Figure 7:**
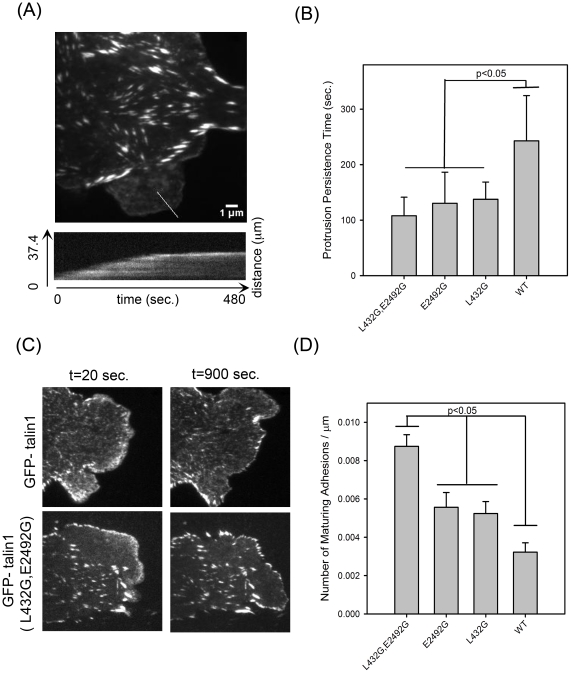
Quantification of the protrusion persistence and number of maturing adhesions in CHO.K1 cells expressing mutants of GFP-talin1. (A) Average fluorescence image and kymograph from a TIRF time series of a CHO.K1 cell expressing GFP-talin 1 (E2492G) plated on 2 µg/ml FN for 20–30 minutes. Images were collected every 0.5 seconds for 8–10 minutes. The kymograph (bottom) of the highlighted protrusion (red line, top) indicates a persistence time of ∼230 seconds. (B) Plot of the persistence times for GFP-talin1, GFP-talin1 (L432G), GFP-talin1 (E2492G), and GFP-talin1 (L432G, E2492G) shows longer protrusion persistence time in cells expressing wild-type GFP-talin1 compared to the single and double point mutants of GFP-talin1. The number of protrusions (Nprot.) and cells (Ncells) included in the analysis was 9,3 (L432G, E2492H); 11,4 (L432G); 9,3 (E2492G); 11,5 (WT) respectively. (C) TIRF images selected from earlier (20 sec.) and later (900 sec.) time points of the above data set. The number and size of adhesions that stabilize and mature as the cell edge protrudes was greater in GFP-talin1 (L432G, E2492G) compared to wild-type GFP-talin1. The average number of adhesions that stabilize per cell edge protrusion is presented in (D). Number of cells (Ncells) included in the analysis was 5 (WT), 6(E2492G), 4 (L432G), 5 (L432G, E2492G). Error bars are SEM. The Holm-Sidak method was used to test the statistical significance of the pairwise differences in the distributions.

We quantified the dynamics of cell protrusions (Fig. 7A, upper panel) and the fraction of nascent adhesions that stabilize and mature at the cell edges (Fig. 7C). The persistence time of a protrusion, which is the time it takes a protruding front to stop and for adhesions to stabilize and grow, varied among the mutants. The GFP-talin1 double (L432G, E2492G) mutant showed the lowest protrusion persistence time (Fig. 7B) and the largest fraction of maturing adhesions per cell front (Fig. 7D), whereas the single point talin1 mutants (L423G) and (E2492G) had intermediate values between wild-type GFP-talin1 and the double mutant. In conclusion, the data indicate that calpain2 cleavage of talin1 at both the N- and C-terminal sites has a significant effect on adhesion dynamics.

In an attempt to detect calpain-mediated cleavage of talin1 in disassembling adhesions at the rear of the cell, we used fluorescence ratio imaging. We compared wild-type talin1 with the double talin1 mutant (L432G, E2492G) dually labelled with GFP and mCherry on opposite ends of the molecule. However, we did not observe any major difference in the ratios between the wild-type talin1 and the double mutant as adhesions disassembled and translocated in retracting regions (Fig. S2). This suggests that either the fraction of cleaved talin1 in disassembling adhesions is small and therefore not readily detected using this approach, or that the N- and C-terminal talin1 fragments are released at the same rate.

## Discussion

We report here the characterisation of a novel calpain2 cleavage site in talin1 that removes the dimerisation helix at the C-terminus of the talin rod. We have identified a mutation (E2492G) that suppresses cleavage at this site in vitro, and show that it leads to an increase in the steady state levels of talin1 in HEK293 cells. Moreover, it reduces FA turnover and cell protrusion in CHO.K1 cells to about the same extent as the well documented L432G mutant that suppresses calpain2 cleavage between the talin head and rod [38]. Importantly, the effects of the point mutations on FA turnover were greatest when both were incorporated into a single talin1 molecule. The results show that calpain2 cleavage of talin1 at both the N- and C-terminal sites is important in regulating FA dynamics.

Recent data suggest that the talin1 head liberated by calpain2 cleavage has a function independent of full-length talin1, and promotes FA turnover [47] and the early phases of cell spreading, including integrin and Src activation, though not FAK signalling or FA assembly [17]. The potential physiological significance of these observations is supported by the finding that the half-life of the talin head is tightly regulated by Smurf1-mediated ubiquitination coupled to proteasomal degradation [47]. The fact that both FA turnover and persistence of cell protrusion is reduced in cells expressing the talin1 L432G calpain2-resistant mutant is consistent with these findings, although it might equally well be explained by an inability to degrade talin and therefore promote FA turnover.

The proposal that the talin1 head has functions independent of full-length talin1 raises the possibility that the talin1 rod might also have its own unique role in the cell. The talin1 rod contains an integrin-binding site and several vinculin- and actin-binding sites (Fig. 1A), and in activated platelets, it is the talin1 rod (not the head) that is incorporated with αIIbβ3-integrin into the TritonX100-resistant cytoskeletal fraction. Wang et al., [54] have recently reported the surprising finding that talin1 knockdown in mammary epithelial cells (which do not express talin2) does not affect cell spreading, integrin activation or the formation of actin stress fibres, but does lead to cell cycle arrest that was attributable to defective FAK signalling. Intriguingly, FAK signalling and cell cycle arrest were rescued by expression of a talin1 rod fragment spanning residues 1974–2541 that contains both the C-terminal integrin- and actin-binding sites (Fig. 1A). While we have been unable to find a calpain2-cleavage site in the talin1 rod that would generate such a fragment, we have identified an internal promoter in the talin2 gene that gives rise to a similar talin rod polypeptide (residues 1608–2543) in kidney, although it is expressed at lower levels in other tissues [14].

The results of Wang et al., [54] strongly suggest that the talin rod has a signalling role that is independent of the talin head. Moreover, they show that the activity of the talin1 1974–2541 rod fragment is dependent on its ability to dimerise via the C-terminal helix. In this scenario calpain2-mediated cleavage of the C-terminal helix might serve to terminate the functions associated with the talin rod polypeptide, although the same mechanism may equally well play a role in inactivating full-length talin, thereby destabilising existing FA. It is notable that the cleavage site between the talin head and rod is more sensitive to calpain2 than that flanking the C-terminal helix. Thus, low levels of active calpain2 may generate sufficient talin head and rod to participate in the integrin activation cycle and FAK signalling respectively, while termination of rod function by removal of the dimerisation domain would require higher levels of calpain activation. While the above data indicate that both the talin head and rod liberated by calpain2 cleavage have independent functions in cell spreading and cell cycle progression, co-expressing the talin1 head and talin1 rod in talin1 knock-down fibroblasts or endothelial cells fails to rescue cell spreading and FA assembly (Kopp et al., unpublished data) indicating that the full-length molecule is essential in this regard.

Calpain2 cleavage of FA proteins at the rear of the cell and its role in tail end retraction and cell migration are well documented [41]. Current data suggest a model in which calpain2 is targeted to and activated at the rear of the cell by a combination of PIP2 (EGF-mediated activation of PLCγ at the leading edge generates a PIP2 gradient) and ERK-mediated phosphorylation on Ser50 [55]. Targeting is rapidly reversed by PKA-mediated Ser369 phosphorylation (induced by the CXCR3 ligands IP-9 and IP-10), which blocks binding of calpain2 to PIP2, and inhibits tail end retraction and cell migration. In this scenario, talin cleavage between the head and rod at the rear of the cell is envisaged to terminate talin function, switch integrins to a lower affinity state, weakening the link between integrins and the actin cytoskeleton and resulting in FA disassembly. However, our ratio imaging data using talin1 tagged at both the N- and C-terminus indicate a low level of cleavage. Since calpain acts on other FA proteins including β3-integrin tails, FAK and vinculin [40], the actual fraction of cleaved talin1 could be small and not detected using our methods. Indeed, mutations that suppress calpain2 cleavage of FAK similarly reduce FA turnover [42], although suppression of calpain2-mediated paxillin cleavage increased FA turnover by an unidentified mechanism [56].

In conclusion, we report here a novel calpain2-cleavage site in talin that removes the C-terminal helix from the talin rod, and show that it is important in FA turnover and cell protrusion. Whether cleavage at this site is regulated in any way has not been explored, although it is interesting to note that phosphorylation of the C-terminal region of filamin by PKC protects against calpain1 cleavage [57].

## Materials and Methods

### Protein expression, purification and calpain2-mediated proteolysis

Mouse talin1 and human talin2 cDNAs were amplified by PCR, cloned into pET-151/D-TOPO (Invitrogen) and authenticated by DNA sequencing. Recombinant talin polypeptides were expressed as His-tagged proteins in E. coli and purified on Ni-NTA (GE Healthcare) columns following standard procedures. The tag was removed by TEV cleavage, and the polypeptides purified further on a Resource-Q column (GE Healthcare). Talin polypeptides were incubated (37°C for 30 minutes) with recombinant rat calpain2 (Calbiochem) in 25 mM HEPES, pH 7.5, 50 mM NaCl, 1 mM DTT ±3 mM CaCl_2_. Analytical gel filtration chromatography of recombinant talin polypeptides was performed using Superdex-75 (10/300) (Amersham Biosciences) at room temperature. The column was pre-equilibrated and run in 20 mM Laflamme Tris pH 8.0, 150 mM NaCl and 2 mM DTT at a flow rate of 0.8 ml/min.

### NMR Spectroscopy and modelling

For NMR experiments, ^15^N-labeled talin polypeptides were transferred into 20 mM sodium phosphate pH 6.5, 50 mM NaCl and 2 mM DTT, 10% (v/v) ^2^H_2_O using a PD10 column (GE Healthcare), and concentrated to 0.15 mM immediately prior to collection of NMR spectra. NMR spectra of all proteins were obtained at 298 K using a Bruker AVANCE AVII 800 spectrometer equipped with a CryoProbe. Spectra were processed with TopSpin (Bruker Corp.) and analyzed using Analysis [58]. The MODELLER [50], [51] software package (version 9v7) was used to construct models of the linker regions between each domain using the align2d and the model-single functions. In the first stage, the known structured regions of talin1 [F3 (PDB ID: 3IVF; [19]); 482–786 VBS1 (PDB ID: 1SJ8 [29]); 2300–2482 ABS3 (PDB ID: 2JSW [27] and 2494–2541 DD (PDB ID: 2QDQ [27]] were aligned with the larger fragments.

### Actin co-sedimentation assays

G-actin was purified from rabbit skeletal muscle and polymerised in 10 mM Tris, 50 mM NaCl, 100 µM ATP, 1 mM DTT, 1 mM MgCl_2_, pH 7.0. Assays were performed using 4 µM talin polypeptides and 10 µM F-actin. The mixture was incubated for 60 min at room temperature and centrifuged at 100,000 rpm for 30 min at 22°C using a Beckman Optima TM ultracentrifuge. Supernatants and pellets were analysed on 12% SDS-PAGE gels and stained using Coomassie blue.

### Cell culture and transfection

The GFP-talin1 and GFP-talin1 L432G expression vectors have been previously described [38]. GFP-talin1 E2492G and GFP-talin1 L432G, E2492G were created by site-directed PCR mutagenesis using the aforementioned vectors. The C-terminal mCherry translational fusions GFP-talin1-mCh and GFP-talin1-(L432G, E2492G)-mCh were created by PCR using the relevant GFP-talin1 construct together with mCherry (a gift from Roger Y. Tsien). All constructs were authenticated by DNA sequencing. mCherry-paxillin has been described previously [58].

To evaluate the stability of the GFP-tagged calpain-resistant talin1 mutants in cells, HEK293 cells (10 cm dishes) were transfected with 5 µg of DNA using Lipofectamine as per manufacturer instructions. For the GFP control 0.2 µg and 5 µg of pcDNA was used. The cells were lysed 24 hours post transfection in 20 mM Tris pH 7.4, 150 mM NaCl, 2 mM EDTA, 0.1% Deoxycholate, 0.5% NP-40 plus 1% protease inhibitor cocktail set III (Novagen). Proteins (20 µg per lane) were resolved by SDS-PAGE and transferred to a nitrocellulose membrane for the Western blotting using anti-GFP antibodies (Roche). Anti-GAPDH (Santa Cruz) was used as a loading control. All experiments were performed in triplicate.

The effects of the calpain-resistant talin1 mutants on FA was studied using CHO.K1 cells (American Type Culture Collection; CCL-61™) cultured in low glucose DMEM supplemented with 10% FBS, 4 mM L-glutamine, 1% (v/v) non-essential amino acids, and penicillin/streptomycin. Cells were maintained in a humidified 5% CO_2_ atmosphere at 37°C. Transfections with GFP-talin1 and paxillin-mCherry DNA or GFP-talin1-mCh and GFP-talin1 (L432G, E2492G)-mCh plasmids were performed using Lipofectamine. CHO.K1 cells were incubated with 5 µl Lipofectamine and 0.1–0.2 µg DNA (supplemented with pBluescript DNA to yield a total of 1 µg DNA) for 3–4 hours. For live time-lapse imaging, cells were trypsinised and replated on fibronectin-coated (2 µg/ml) glass bottom dishes in CCM1 medium (HyClone, Logan, UT). Cells were maintained at 37°C using a Warner Instruments heated stage insert (Bioptechs, Butler, PA; Warner Instruments, Hamden, CT).

#### Fluorescence Microscopy and Image Analysis

TIRF image time series were acquired on an Olympus IX71 microscope using a 100×1.45 NA Plan Apo TIRFM oil objective, a Ludl controller (Ludl Electronic Products, Hawthorne, NY), and Metamorph Software (Molecular Devices, Downingtown, PA). The 488 nm and 568 nm lines of an Ar-Kr ion laser (Melles Griot, Albuquerque, NM) were used to excite fluorescence from GFP- and mCherry-tagged constructs respectively. Laser powers were modified and monitored by an acousto-optical tunable filter (AOTF) unit with digitized power readout (LSM technologies, Etters, PA). For simultaneous dual-colour imaging, a polychroic mirror (Z488/568 rpc), dual emission filter (Z488/568 nm) (Chroma Technology, Bellows Falls, VT), and Dual-View (Photometrics, Tucson, AZ) were used. Image time series were acquired using the QuantEM: 512C EMCCD camera (Photometrics, Tucson, AZ). Quantification of adhesion size and dynamics were performed in Image J (MBF-ImageJ for Microscopy, McMaster University, Hamilton, Ontario, Canada). Images were corrected for detector and diffuse background fluorescence. A segmentation algorithm using maximum entropy threshold was used to isolate and measure the size of adhesions from images of GFP-talin1 mutants in CHO.K1 cells. We used the GFP-talin1 channel for adhesion size quantification since variations in fluorescence intensity and adhesion size between the GFP-talin1 and paxillin-mCherry channels were minimal and constant among the different talin1 mutants. Kymography analysis was implemented on image time series to measure the persistence time of cell edge protrusions. We define the persistence time as the time at which a protrusions stop and nascent adhesions mature and grow. For ratio imaging analysis of dual labeled GFP-talin1-mCh and GFP-talin1-(L432G, E2492G)-mCh, cells were corrected for background intensity effects and the channel 1 (GFP) was divided by channel 2 (mCherry) to yield a ratio coefficient (R). For statistical comparison analysis, we use the Kruskall-Wallis ANOVA on ranks approach. Multiple Pairwise comparisons were performed in Sigma Plot 11.0 following the Dunn's method and the Holm-Sidak method for data presented in Figures 6 and 7 respectively.

## Supporting Information

Figure S1 **Epitope mapping of the monoclonal antibody TD77.** (A) The recombinant talin polypeptides (2.5 µg) indicated were analysed by SDS-PAGE and stained with Coomassie blue. (B) Western blot analysis of the talin proteins (100 ng) using TD77 (1∶5000) reveals the residues L2515 and R2526 as essential for TD77 recognition. (C) Cartoon of the talin dimerisation domain showing the relative positions of the key residues (L2515 and R2526) of the TD77 epitope.
(TIF)

Figure S2 **Fluorescence ratio imaging of wild-type and (L432G,E2492G) calpain resistant talin1 in retracting adhesions in CHO.K1 cells.** Image time series of cells expressing GFP-talin1-mCh (left) or GFP-talin1 (L432G, E2492G)-mCh (right) were collected every 5 sec for 10 min. (A) Images of summed time series for both construct highlight the location and direction of the retracting adhesions (boxed regions). Intensity time traces and ratios (R) for selected pixels along the highlighted retracting adhesions in (A) for GFP-talin1-mCh (B) and GFP-talin1 (L432G, E2492G)-mCh (C). The ratio is channel 1(GFP-black) divided by channel 2 (mCh-red).
(TIF)
