# Supplementary figures and images for: Talin Contains A C-Terminal Calpain2 Cleavage Site Important In Focal Adhesion Dynamics
Source: PLoS One. 2012 Apr 4;7(4):e34461. doi: 10.1371/journal.pone.0034461 (PMC3319578; doi:10.1371/journal.pone.0034461)

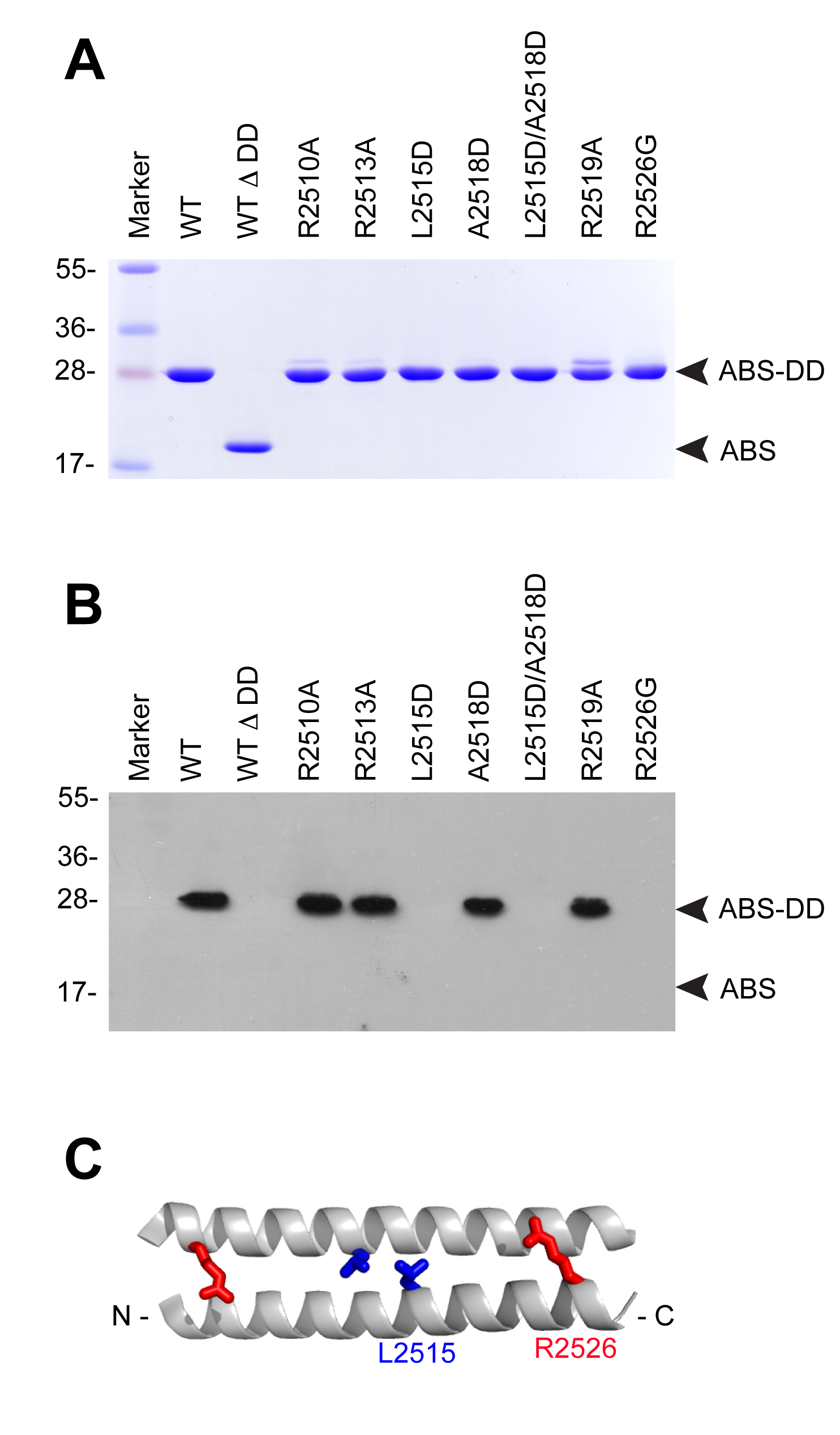

Supplement: Figure S1 — Epitope mapping of the monoclonal antibody TD77. (A) The recombinant talin polypeptides (2.5 µg) indicated were analysed by SDS-PAGE and stained with Coomassie blue. (B) Western blot analysis of the talin proteins (100 ng) using TD77 (1∶5000) reveals the residues L2515 and R2526 as essential for TD77 recognition. (C) Cartoon of the talin dimerisation domain showing the relative positions of the key residues (L2515 and R2526) of the TD77 epitope. (TIF) [file pone.0034461.s001.tif]

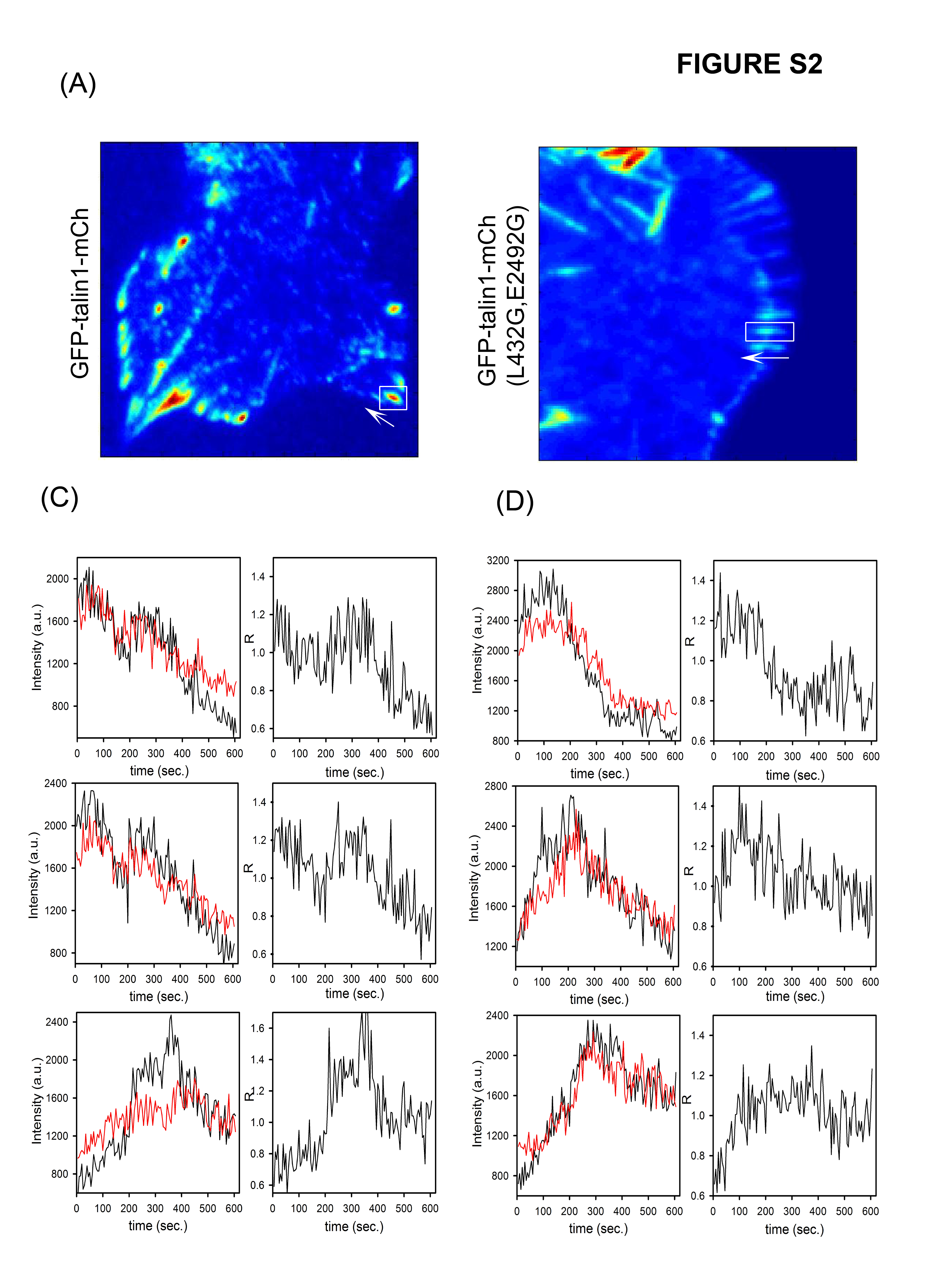

Supplement: Figure S2 — Fluorescence ratio imaging of wild-type and (L432G,E2492G) calpain resistant talin1 in retracting adhesions in CHO.K1 cells. Image time series of cells expressing GFP-talin1-mCh (left) or GFP-talin1 (L432G, E2492G)-mCh (right) were collected every 5 sec for 10 min. (A) Images of summed time series for both construct highlight the location and direction of the retracting adhesions (boxed regions). Intensity time traces and ratios (R) for selected pixels along the highlighted retracting adhesions in (A) for GFP-talin1-mCh (B) and GFP-talin1 (L432G, E2492G)-mCh (C). The ratio is channel 1(GFP-black) divided by channel 2 (mCh-red). (TIF) [file pone.0034461.s002.tif]
